# Remodeling of Metabolic and Secretory Organelles During Oncogenic and Oncomodulatory Viral Infections

**DOI:** 10.3390/v18030288

**Published:** 2026-02-27

**Authors:** William Rodriguez, Ileana M. Cristea

**Affiliations:** 210 Lewis Thomas Laboratory, Department of Molecular Biology, Princeton University, Princeton, NJ 08544, USA; wr6263@princeton.edu

**Keywords:** KSHV, EBV, HBV, HCV, HPV, HTLV-1, MCPyV, HCMV, virus, organelle remodeling

## Abstract

Persistent oncovirus infections account for 15–20% of the global cancer burden, driving multiple forms of human cancer. To maintain persistent infection and spread, oncoviruses drive alterations in host cell metabolism, immune signaling, and cell-to-cell communication throughout tumor microenvironments. Accumulating evidence has indicated that these alterations occur in conjunction with a range of organelle remodeling events that can differ between “dormant” viral latency and active lytic replication. Throughout each phase of infection, oncoviruses alter the morphology, composition, and function of organelles to promote cellular survival and proliferation, while periodically supporting viral replication. Here, we review oncovirus-driven organelle remodeling strategies across distinct infection states, including viral latency, reactivation from latency, and chronic active replication. We focus on the molecular mechanisms by which oncovirus-driven organelle remodeling promotes cellular transformation, impedes immune responses, and facilitates virion assembly and egress. We also draw parallels between remodeling strategies employed by oncogenic and oncomodulatory viruses, emphasizing broadly conserved mechanisms across cancer-associated infections. Lastly, we highlight how studies of oncovirus organelle remodeling are critical for discovering vulnerabilities in both oncogenic virus infection and viral oncogenesis, with therapeutic potential for multiple cancers.

## 1. Introduction

A persistent challenge of the current era is the public health threat and socioeconomic burden of cancer. Viral infections contribute significantly to this burden, accounting for approximately 1.4 million cancer cases annually—15–20% of global cases [1]. Virus-linked cancers are primarily driven by seven oncogenic viruses (oncoviruses): Kaposi’s sarcoma-associated herpesvirus (KSHV), Epstein–Barr virus (EBV), hepatitis B virus (HBV), hepatitis C virus (HCV), human papillomavirus (HPV), human T-cell lymphotropic virus type-1 (HTLV-1), and Merkel cell polyomavirus (MCPyV) [2]. Oncomodulatory viruses, such as human cytomegalovirus (HCMV), herpes simplex virus-1 (HSV-1), and human immunodeficiency virus (HIV-1) also establish infections that increase the risk for cancer development [3,4,5,6]. A major challenge in treating virus-linked cancers is that most arise years to decades after the primary infection, largely due to the ability of these viruses to establish lifelong infections that evade immune clearance. These long-term infections can take several forms, including the establishment of viral latency, where the viral genome replicates in synchrony with the host, with minimal viral gene expression [7,8,9,10,11]. Oncoviruses can reactivate from latency multiple times throughout an individual’s lifespan, especially during immunosuppression, hormonal changes, or co-infections with other viruses, with this reactivation promoting virus replication and spread [12,13,14,15,16]. Alternatively, other oncoviruses persist through chronic active replication, maintaining a localized site of infection that also promotes cellular transformation across a tissue environment [17,18]. Understanding how oncoviruses support either the biphasic latent–active replication cycle or chronic active replication continues to be a critical challenge in the development of therapeutic strategies.

Over 15 distinct cancers are linked to oncovirus infections and their multiple tissue tropisms, with diverse host, viral, and environmental factors contributing to viral oncogenesis [1,2]. The dsDNA viruses KSHV and EBV establish latent infections across multiple cell types, promoting different cancers. These include Kaposi’s sarcoma (KS) from KSHV infection of endothelial and potentially mesenchymal stem cells [19], nasopharyngeal carcinoma (NPC) and gastric carcinoma (GC) from EBV-latent epithelial cells [20,21], and multiple B-cell lymphomas, such as KSHV and EBV-driven primary effusion lymphoma (PEL) and EBV-driven Burkitt’s lymphoma (BL) [22]. For EBV, different cancers arise depending on latent gene expression patterns across Type III, Type II, Type I, and Type 0 latency [15], which exhibit progressively restricted gene expression [23]. Additionally, oncovirus infection of cervical epithelium by HPV, T-lymphocytes by HTLV-1, dermal fibroblasts by MCPyV, and hepatocytes by HBV and HCV can lead to cervical cancer [24], adult T-cell leukemia/lymphoma (ATL) [25], Merkle cell carcinoma (MCC) [16], and hepatocellular carcinoma (HCC) [26], respectively. These observations demonstrate how oncoviruses have evolved to modify diverse cell types to support persistent infection through viral latency or chronic active replication. Infection outcomes also include indirect transformation of neighboring uninfected cells across the viral tumor microenvironment through excessive oxidative stress and inflammatory responses [27,28,29]. Despite oncoviruses’ significant contribution to the global cancer burden, much remains to be understood as to how host cells are altered to support latent or active infection across diverse cell types, and how these modifications drive cancer progression.

A universal feature of viral infection is reliance on organelle remodeling to tune organelle functions and inter-organelle communication to support viral replication [30]. Modulation of organelle biology is critical for different steps of the virus life cycle, with alterations in organelle morphology ranging from subtle shifts in host membrane composition and architecture to the formation of infection-specific subcellular structures. In parallel to pro-viral remodeling, organelle alterations can also serve as host antiviral responses, supporting the activation of immune signaling and stress responses to restrict viral replication and spread. Studies across multiple viral families have started to link infection-driven organelle remodeling to distinct infection and/or pathogenesis outcomes [31,32,33,34,35,36]. Human oncoviruses similarly rewire organelle dynamics to support both active viral replication and long-term cell survival and proliferation during viral latency. Importantly, accumulating evidence has pointed to links between organelle remodeling events—within or outside the context of viral infection—and hallmark cancer phenotypes, including genetic instability [37,38,39,40,41,42,43,44], reprogramming of cellular metabolism and oxidative stress [45,46,47,48,49,50,51,52,53], and alterations of lipid flux [47,48,54,55,56,57,58]. These findings emphasize the need for additional studies on how oncoviruses modify organelle structure–function relationships and how these remodeling events contribute to viral oncogenesis.

In this review, we discuss links between the outcomes of infections by oncogenic viruses and alterations in organelle number, morphology, and composition. We consider the current knowledge of organelle remodeling during both latent and active viral infections. Through these two lenses, we highlight molecular mechanisms by which oncoviruses drive pro-viral organelle remodeling events and whether these changes have been linked to infection outcomes and/or cancer progression. We do not discuss MCPyV-linked organelle remodeling in this review, as there is still limited knowledge in this research area. We focus our discussion on infection-driven remodeling of mitochondria, peroxisomes, lipid droplets, and secretory compartments (ER, Golgi, and endolysosomes) for modulation of cellular metabolism, immune responses, and support of virion assembly and egress.

## 2. Organelle Remodeling During Latent Oncovirus Infection

**Figure 1:**
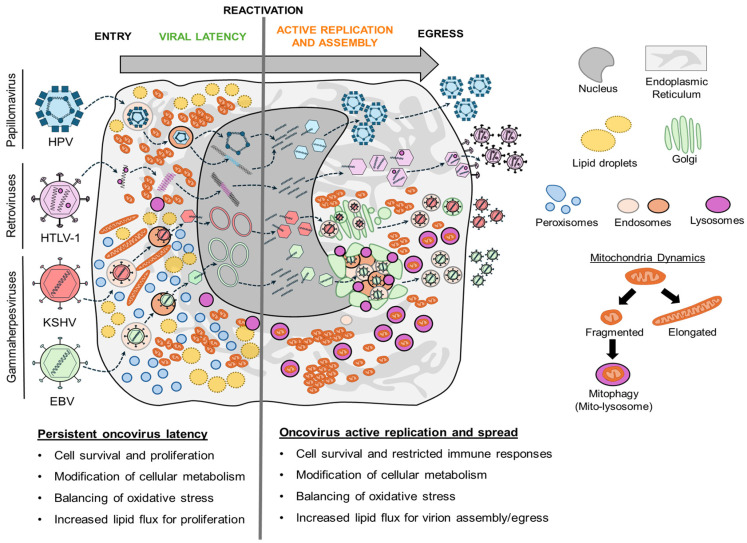
Oncovirus latency and active replication induce organelle remodeling with connections to viral oncogenesis: Biphasic oncovirus infections remodel organelle structure and function. This schematic shows the replication cycles of human papillomavirus (HPV) (**top**), human T-lymphotropic virus (HTLV-1) (second from **top**), Kaposi’s sarcoma-associated herpesvirus (KSHV) (second from **bottom**), and Epstein–Barr virus (EBV) (**bottom**). The illustrations depict how these viruses alter organelle structure/organization during viral latency and following reactivation into active replication. Primary infection, latency establishment, reactivation, assembly, and egress are represented from left to right. A legend is provided at the right-hand side for identifying each individual organelle, as well as distinct mitochondria remodeling occurring across infection states for these four viruses. Below the schematic are documented outcomes of viral oncogenesis linked to oncovirus-induced organelle remodeling during both states of infection.

Viral latency is a “dormant” infection state where viral genetic material is retained and replicated passively with each host cell division without de novo virion production [59]. Latency features limited or absent viral gene expression, allowing oncoviruses to evade immune surveillance. Through latency, oncoviruses such as KSHV, EBV, HPV, HTLV-1, and MCPyV establish life-long infections that can promote cancer progression when unrestricted by immune responses [2,60,61]. Cellular transformation and viral genome maintenance during latency collectively require alterations to fundamental cellular processes. These include modifications of central metabolism, oxidative stress, and lipid flux. Throughout this section, we discuss how latent oncovirus infections remodel mitochondria, peroxisomes, and lipid droplets to meet the metabolic requirements of viral latency and sustain cell survival and proliferation (Figure 1, Table 1).

### 2.1. Mitochondria Remodeling During Viral Latency

Amongst the host cell alterations induced by viral latency, one of the best documented is metabolic dysregulation, where oncovirus infection shifts the balance from oxidative phosphorylation (OXPHOS) to glycolysis. Like many viral and non-viral cancers, oncovirus-transformed cells often exhibit increased reliance on glycolysis over mitochondrial OXPHOS despite oxygen availability, a metabolic state termed “aerobic glycolysis” or “Warburg metabolism” [62,63,64]. This glycolytic shift results in increased lactate production and secretion, which has been shown to inhibit immune signaling [65,66,67]. Aerobic glycolysis is often paired with changes in glutaminolysis and fatty acid metabolism, all supporting accelerated biomolecule production for sustained cellular proliferation [68,69,70,71]. One mechanism driving these glycolytic shifts across oncovirus infections is infection-driven changes in mitochondrial form and function. Mitochondria regulate multiple fundamental processes, including OXPHOS, reactive oxygen species (ROS) levels, calcium homeostasis, immune signaling, and programmed cell death, especially apoptosis and ferroptosis [72,73,74,75]. This functional breadth reflects mitochondrial plasticity, i.e., their ability to dynamically alter morphology, distribution, and cellular contacts in response to environmental stimuli. These processes are coordinated by several proteins regulating mitochondrial fission (Drp1, Fis1, Mff), fusion (Mfn1, Mfn2, Opa1, Opa2), and mitophagy (Parkin, Pink1, LC3B), which we and others have shown to be modulated during viral infections [49,53,76,77,78,79,80,81]. Given their vital roles, mitochondria are frequently targeted during viral infections for control of glucose, lipid metabolism, and innate immunity [30]. Here, we highlight mechanisms by which latent oncovirus infection alters mitochondrial dynamics and how these remodeling events affect cellular metabolism, oxidative stress, survival, and proliferation.

#### 2.1.1. Mitochondria Remodeling During KSHV Latency

During KSHV latency, the virus expresses 12 viral microRNAs (miRNAs) from its latency locus [82] that are essential for KSHV-induced tumorigenesis [83,84,85,86,87]. These miRNAs promote cellular transformation by shifting lymphatic endothelial cell (LEC) metabolism from oxidative phosphorylation to aerobic glycolysis through HIF1α activation [86]. This metabolic shift results in part from viral miRNA-mediated silencing of genes critical for mitochondrial biogenesis and protein import, thereby reducing mitochondrial numbers. This reduction in mitochondria numbers suggests that KSHV infection alters mitochondrial function to support viral latency. Emphasizing this link between mitochondria remodeling and latency maintenance, treatment of KSHV-latent endothelial and PEL B cells with resveratrol, an inducer of mitochondrial biogenesis, significantly reduces cell viability and triggers spontaneous KSHV lytic reactivation [86]. These findings demonstrate that KSHV miRNA-mediated alterations in mitochondrial biogenesis and metabolism impact latent cell survival and are involved in maintaining the balance between KSHV latency and active lytic replication.

Alongside viral miRNAs, KSHV encodes several viral latency proteins, including LANA (ORF73), viral FLIP (vFLIP), viral cyclin (vCyclin), and several Kaposins (KapA-C), that each contribute to KSHV tumorigenesis [87]. Like the KSHV miRNAs, a CRISPR-Cas9 knockout screen of the human genome by Holmes and colleagues identified host and viral factors that interface with mitochondria and may contribute to latency-driven alterations of mitochondrial dynamics [88]. This screen pointed to approximately 146 host factors as being essential for KSHV-latent endothelial cell survival or proliferation, with a subset of these factors being localized to mitochondria and functioning in mitochondrial translation and respiration. Accordingly, targeted perturbation of mitochondrial translation, respiration, or mtDNA integrity negatively impacts survival and proliferation of KSHV-latent endothelial cells and two KSHV-positive PEL B-cell lines. Just as reported in LECs, KSHV latency in telomerase-immortalized endothelial (TIME) cells significantly alters mitochondrial morphology, increasing mitochondria length and decreasing their numbers, suggesting enhanced mitochondrial fusion [88]. These alterations appear to be coordinated by several of the above-mentioned viral latency proteins—vFLIP, vCyclin, and KapB—that all localize to mitochondria in KSHV-latent endothelial cells. Thus, KSHV latency can also directly remodel mitochondrial form and composition to support latent cell survival and proliferation.

#### 2.1.2. Mitochondria Remodeling During EBV Latency

Like KSHV, EBV has evolved to modulate cellular metabolism through the function of several latency proteins, including two viral oncoproteins, LMP1 and LMP2A [11]. LMP2A, a plasma membrane-bound latency protein, is sufficient to induce cellular transformation, anchorage-independent growth, and invasiveness by driving the epithelial-to-mesenchymal transition (EMT) [89,90,91]. During EMT, epithelial cells gain mesenchymal cell traits, including increased invasion ability and resistance to apoptosis [92]. Pal and colleagues found that LMP2A expression in AGS gastric carcinoma cells (GC) induces mitochondrial fission by promoting the phosphorylation of the fission factor Drp1 at Ser616, while restricting expression of the fusion factor Mfn1 [93]. LMP2A-induced Drp1 expression directly enhances epithelial cell migration, linking mitochondrial fragmentation to metastatic phenotypes observed across multiple non-viral cancers, including breast, ovarian, and pancreatic cancers [94]. LMP2A activates Drp1 through the Notch signaling pathway, which induces EMT markers via crosstalk between PI3K and AKT signaling cascades. Notably, Notch signaling can also increase glycolysis and lactate production characteristic of Warburg metabolism [95,96,97], suggesting that LMP2A-induced mitochondrial fragmentation through Notch signaling may represent another pathway for inducing glycolysis during viral latency [98].

Paralleling LMP2A activation of Drp1 in EBV-latent gastric carcinoma cells [93], Xie and colleagues found that LMP1 expression in nasopharyngeal carcinoma (NPC) (Type II latency) also induces mitochondrial fission [99]. LMP1 expression in NPC patient tissues correlated with increased Drp1 phosphorylation at Ser616 and decreased inhibitory phosphorylation at Ser637. Supporting this, EBV latent infection and LMP1 expression alone in NPC cell lines promote mitochondrial fragmentation through Drp1 (Ser616) activation. Furthermore, Drp1 inhibition with the small molecule Drp1 inhibitor Mdivi-1 impaired EBV-latent cell survival and proliferation by inducing necroptosis and apoptosis [100]. While LMP1 promotes Drp1 function by suppressing inhibitory Drp1 phosphorylation (Ser637) through repression of AMPK activity, it also induces Drp1 activation by promoting the expression of the cyclin B1/CDK1 complex. Remarkably, drug inhibition of either pathway—using metformin for AMPK or cucurbitacin for cyclin B1/CDK1—combined with cisplatin [101], overcame NPC chemoresistance in vivo. This study highlights how gammaherpesvirus latency-induced oncogenic stress can indirectly alter mitochondrial dynamics.

While EBV has evolved to target the mitochondrial fission/fusion machinery, remodeling of mitochondria morphology can also be achieved through dysregulation of lipid synthesis pathways. A recent study by You and colleagues found that EBV transformation of lymphoblastoid cell lines (LCLs) (involving Type II and Type III latency transitions) significantly increases cardiolipin biosynthesis [34], a phospholipid essential for mitochondrial cristae biogenesis. The EBV latency protein EBNA2 induces this cardiolipin increase through a MYC-dependent activation of cardiolipin biosynthesis enzymes, leading to increased cristae length and number per mitochondrion. Cardiolipin biosynthesis inhibition impedes EBV-transformed LCL viability and alters mitochondrial respiration. Decreased cardiolipin biosynthesis also disrupts EBV enhanced one-carbon metabolism and aspartate biosynthesis by destabilizing critical mitochondrial complexes. These results reveal how EBV latency and B-cell transformation rely on cardiolipin to remodel the mitochondrial ultrastructure, thereby promoting metabolic changes that support latent cell survival and proliferation.

#### 2.1.3. Mitochondria Remodeling During HPV Latency

Following primary infection of cervical basal epithelial cells, HPV establishes viral latency as an episome or by integrating into the host genome [24]. During latency, HPV minimally expresses several genes from the early (E) region to coordinate genome replication and promote cellular transformation [102]. To support this process, HPV infection can alter multiple features of mitochondrial composition, function, and turnover, including mtDNA integrity [36,103,104], mitophagy [105,106], mitochondrial ROS (mtROS) production [107,108,109,110], and mitochondria-mediated apoptotic pathways [111]. There are also several reports demonstrating that HPV E proteins can either directly or indirectly modify mitochondrial structure–function relationships. One of the earliest of these studies by Lai and colleagues found that high-risk HPV genotype 18 (HPV-18) E2 protein promotes mitochondrial fission [112]. Unlike most latency proteins discussed throughout this review, E2 is well-documented for anti-proliferative functions, silencing E6 and E7 expression by binding the long control region promoter [113] and inducing apoptosis independently [114]. However, reports now suggest high-risk HPV E2 has more nuanced, context-specific oncogenic functions, including inducing cell cycle arrest [115] and promoting skin cancer in mouse models [116]. Lai and colleagues showed that HPV-18 E2 accumulates in the nucleus until reaching a threshold concentration, followed by its translocation to the mitochondrial inner membrane (IMM), where it interacts with complexes III and IV (COX-III and COX-IV) and ATP synthase [112]. Electron microscopy has revealed that HPV-18 E2 expression, but not HPV-6 E2, significantly disrupts cristae structure and increases mtROS production. Despite these mitochondrial alterations, apoptosis was not induced, suggesting that alternative ROS management mechanisms may be activated by E2 to compensate. Paralleling these changes in mitochondrial structure and ROS production, HPV-18 E2 expression also stabilizes HIF1α under normoxic and hypoxic conditions, pushing cells toward a Warburg metabolism, as evidenced by increased lactate secretion in a HIF1α-dependent manner. Additionally, E2 proteins from low-risk HPV strains could not drive similar mitochondria remodeling, linking HPV-induced mitochondrial remodeling to poor prognosis in infection-related cervical cancers [117].

Several other high-risk HPV early proteins also directly or indirectly interface with mitochondria, altering their structure and function. HPV-16 E4 detaches mitochondria from the cytoskeletal network, reduces membrane potential, and sensitizes cells to apoptosis [118]. HPV-16 and HPV-18 E6 increase mitochondrial OXPHOS by modulating electron transport chain complex expression, induce mtROS accumulation, and trigger DNA damage that supports HPV transformation [119]. Notably, while E6 induces mtROS accumulation, oxidative stress, and DNA damage, it does not trigger apoptosis, likely because E6 decreases p53 levels and restricts mitochondrial cytochrome C release [120,121]. Additionally, Thomas and colleagues found that HPV-16 E7 enhances ceramide-mediated lethal mitophagy and mitochondrial fragmentation through Drp1 activation in response to cisplatin in an HPV-positive head and neck squamous cell carcinoma (HNSCC) model [105]. Collectively, these studies highlight multiple mechanisms by which HPV early proteins remodel mitochondrial form and function to support oncogenic phenotypes that likely play diverse roles throughout the HPV replication cycle across the cervical epithelium.

#### 2.1.4. Mitochondria Remodeling During HTLV-1 Latency

Unlike HPV, where integration is an accidental feature of persistent infection, the retrovirus human T-lymphotropic virus-1 (HTLV-1) actively integrates its genome into the host genome as a provirus during latency [122]. Following integration in CD4+ T cells, HTLV-1 expresses limited viral genes sufficient to drive cellular transformation [123]. One latency maintenance protein is p13 (HTLV-1 ORFII), which localizes to and alters mitochondrial function [124,125,126]. p13 is known to localize to the inner-mitochondrial membrane in epithelial and T cells [125] and to induce abundance-dependent mitochondrial alterations. Low p13 levels alter inner membrane potassium (K^+^) permeability and increase OXPHOS activity, while high p13 levels depolarize mitochondrial membrane potential and increase fragmentation. These phenotypes are attributed to four arginine residues forming the charged face of the p13 mitochondrial targeting signal [125,127]. Interestingly, p13-induced potassium (K^+^) influx increases mitochondrial ROS production, sensitizing cells to apoptosis following Fas ligand exposure in T cells [128]. These results suggest that, in contrast to mitochondrial modifications during KSHV and EBV latency, HTLV-1 p13-induced mitochondrial changes oppose HTLV-1-mediated cellular transformation, potentially restricting cell survival and proliferation in vivo. This is intriguing given that p13 can interfere with the functions of HTLV-1 Tax, a transactivation protein that initiates active viral replication, inhibiting Tax-mediated transformation through NF-κB and CREB signaling [35].

### 2.2. Peroxisome Remodeling During Latent Oncovirus Infection

During oncovirus latency, regulation of lipid metabolism and redox homeostasis pathways are essential for latent cell survival and proliferation. Proliferating latent cells have high metabolic demand for lipid synthesis, especially for plasma membrane production and remodeling for pro-viral cell signaling. This is exemplified across viral and non-viral tumors, where studies have shown increased rates of very long chain fatty acid (VLCFA) and ether lipid metabolism [129,130,131]. Lipid metabolism pathways also serve as ROS sinks, making them critical for cancer cell redox balance [132]. To regulate lipid flux and oxidative stress, several oncoviruses alter the peroxisomal compartment. Peroxisomes are single-membrane organelles involved in expanding enzymatic processes, including VLCFA and ether phospholipid (e.g., plasmalogen) metabolism, as well as in ROS reduction through peroxisomal catalase [133]. Given these critical functions, peroxisomes have gained attention as major contributors to cancer progression [134], with studies linking altered peroxisomal protein abundances to poor prognosis in breast cancer, melanoma, renal carcinoma, and hepatocellular carcinoma [135,136,137,138]. In this section, we highlight mechanisms by which latent oncovirus infection alters peroxisome dynamics and how this organelle contributes to lipid metabolism, supporting oncovirus latency.

#### 2.2.1. Peroxisome Remodeling During KSHV Latency

In 2017, Sychev and colleagues showed that KSHV latency in endothelial cells increases the abundance of proteins involved in peroxisome fatty acid metabolism (SCP2, ACSL3, MLYCD, AGPS, EHHADH) and biogenesis (PEX19, PEX12, PEX5) [139]. ABCD3 (PMP70), a membrane-bound peroxisome lipid transporter, is also significantly increased in three KSHV-latent endothelial cell lines, coinciding with increased levels of other peroxisome factors (PEX3, MLYCD, PEX19) that coordinate the biogenesis of this organelle. Indeed, these changes corresponded to a KSHV latency-induced increase in peroxisome numbers [139]. The KSHV latency locus alone is sufficient to drive increased peroxisome numbers and elevated lipid metabolites both upstream and downstream of peroxisomal enzymatic function. ACOX1 knockdown, which impairs the peroxisome beta-oxidation of these lipids, selectively decreased KSHV latent endothelial cell viability. Similarly, ABCD3 knockdown, which impairs docosahexaenoic acid (DHA) import into peroxisomes, significantly reduced latent cell viability. These findings suggest that peroxisome function is important for KSHV-latent endothelial cell survival, and that DHA production and likely other polyunsaturated fatty acids (PUFAs) may support latency maintenance. The latency-induced increase in peroxisome numbers, while mediating these critical steps in DHA metabolism, could also provide a signaling platform for the functions of KSHV latency proteins. Supporting this, Choi and colleagues found that KSHV vFLIP, a viral oncoprotein [140,141,142], localizes to peroxisomes in a PEX19-dependent manner where it is stabilized by MAVS complexes and supports latent cell viability [143]. These findings demonstrate that KSHV latency not only alters peroxisome numbers but also exploits the peroxisomal immune signaling axis to support viral latency.

#### 2.2.2. Peroxisome Remodeling During EBV Latency

Reinforcing the importance of peroxisomes during latent gammaherpesvirus infection, EBV transformation of LCLs also alters peroxisomal protein abundances and concentrations of several peroxisome-metabolized lipid species, such as the VLCFAs C26:0, C24:0, C22:0, and C16:0 [144]. Surprisingly, while peroxisomal proteins involved in beta-oxidation and biogenesis were upregulated during EBV transformation, ABCD1 and ABCD2 lipid importer expression were downregulated by 1–2 days post-infection (dpi). This suggests that EBV latency establishment prevents VLCFA import into peroxisomes. Concomitantly, genes involved in VLCFA synthesis (e.g., ELOVL1) increased, while genes regulating VLCFA degradation decreased, explaining the observed VLCFA accumulation. Further exacerbating this phenotype is EBV induction of the host miRNAs miR-9-5p and miR-155, which repress expression of the peroxisome lipid importers ABCD1 and ABCD2 [144]. This highlights how oncoviruses can take advantage of host miRNAs as opposed to relying solely on viral miRNA expression. ABCD1 restriction was not limited to EBV, also occurring upon infection with other herpesviruses (HSV-1, VZV, HCMV, roseolovirus, KSHV) and coronaviruses (MERS-CoV, SARS-CoV-1, SARS-CoV-2), pointing to a broader relationship between viral infection and peroxisomal lipid import. Supporting the importance of peroxisomal beta-oxidation during EBV infection, treatment of EBV-immortalized B cells with a cholesterol metabolite, 25-hydroxycholesterol (25-HC) [145], enhances beta-oxidation rates, reduces VLCFA levels, and even restores ABCD1 expression. Surprisingly, this induction of beta-oxidation through 25-HC also significantly impairs EBV lytic replication following reactivation from latency. These results demonstrate that EBV latency establishment increases VLCFA synthesis while impairing peroxisomal import and beta-oxidation. These alterations of peroxisome function have direct consequences for both EBV latency maintenance and as a preparatory step before reactivation into lytic replication. Indari and colleagues further supported these findings, showing that EBV latency in two Burkitt’s lymphoma models differentially regulated multiple peroxisomal proteins, beta-oxidation proteins, and cholesterol/ether lipid synthesis compared to EBV-negative B cells [146].

### 2.3. Lipid Droplet Remodeling During Latent Oncovirus Infection

While peroxisomes maintain lipid flux and redox homeostasis, lipid droplets share these roles by providing compartments for lipid storage and metabolism. Lipid droplet accumulation occurs across viral and non-viral cancers, promoting cancer cell survival and proliferation. This effect is linked to several lipid droplet functions by: (1) providing free fatty acids and substrates for membrane synthesis, invasion, and metastasis [147], (2) mediating PLIN protein-mediated TCA cycle activation through HIF2α induction [148], and (3) alleviating ER stress by sequestering and shielding PUFAs from ROS species, thereby evading cell death pathways including ferroptosis [149,150,151]. The lipid droplet number and morphology during oncovirus infection are altered by infection-driven changes in lipid synthesis pathways, including the expression of ACAT and DGAT proteins that synthesize triacylglycerols (TAGs) and cholesterol esters forming the lipid droplet core at the ER surface [152,153,154]. In this section, we highlight mechanisms by which latent oncovirus infections alter lipid droplet dynamics and how this compartment contributes to fatty acid storage and metabolism alterations required for latency maintenance.

#### 2.3.1. Lipid Droplet Remodeling During KSHV Infection

KSHV latency activates fatty acid synthesis pathways and increases lipid droplet numbers across multiple infection models [155,156,157,158]. Delgado and colleagues showed that KSHV latency in endothelial cells elevates host lipogenesis rates, long-chain fatty acid synthesis, and lipid droplet formation [155]. Inhibiting fatty acid synthesis using TOFA (an ACC1 inhibitor) or C75 (a fatty acid synthase (FASN) inhibitor) selectively induces apoptosis in KSHV-latent endothelial cells compared to uninfected cells. This phenotype is rescued by palmitic acid, an ACC1-produced metabolite, suggesting the survival of KSHV-latent cells relies on fatty acid metabolism products coordinated by lipid droplet accumulation and localization. Notably, this lipid droplet increase was observed in mixed cultures of latent and lytic TIME cells, so the contribution of active KSHV lytic replication to lipid droplet accumulation remains unclear. Angius and colleagues showed that KSHV lytic replication following primary infection and latency establishment also increases lipid droplet numbers, with alterations to cholesterol ester and triglyceride synthesis pathways [156]. Indeed, cholesterol ester synthesis inhibition using SZ 58035 significantly impaired neo-angiogenic activity of KSHV-infected endothelial cells throughout both infection phases, a major determinant of Kaposi’s sarcoma progression [159].

#### 2.3.2. Lipid Droplet Remodeling During EBV Infection

Paralleling KSHV studies, EBV latency or LMP2A expression alone significantly increases lipid species levels and lipid droplet numbers in nasopharyngeal carcinoma (NPC) cells [98]. When lipid synthesis was inhibited with 1-butanol, LMP2A cells showed reduced migration capacity, linking lipid synthesis and lipid droplet accumulation to LMP2A-induced invasiveness and metastasis in NPC cells. LMP2A also downregulates ATGL, which breaks down lipid droplet triglycerides. ATGL restriction by LMP2A correlates with increased migratory capacity in LMP2A-positive NPC cells and marks poor prognosis in NPC patient samples. Similarly, Hulse and colleagues demonstrated that EBV transformation of primary B cells induces lipid droplet accumulation in an LMP1-dependent manner [160]. This lipid droplet increase correlates with LMP1-induced FASN stabilization through ubiquitin-specific protease USP2a, which increases lipogenesis. Like LMP2A in NPC cells, USP2A inhibition—and thereby FASN destabilization—impaired survival of EBV-latent Mutu III (Type III latency), Mutu I (Type I latency), and EBV-transformed primary LCLs. This phenotype is rescued by the reintroduction of the FASN metabolite palmitic acid. These studies emphasize that lipid flux and storage are critical for EBV latent cell survival, and that modified fatty acid synthesis and subsequent lipid storage are clear requirements for herpesvirus-linked cancers.

#### 2.3.3. Lipid Droplet Remodeling During HPV Latency

Alongside HPV-encoded E6, HPV E7 protein is best documented for its roles in promoting HPV-mediated cellular transformation through metabolic reprogramming at multiple interfaces including lipid metabolism [161,162]. Reinforcing its capacity for transformation, Darimont and colleagues demonstrated that high-risk HPV-16 E7 protein transduced alongside human telomerase reverse transcriptase (hTERT) can immortalize a human white preadipocyte cell line [163]. E7 expression enhances expression of several transcription factors such as peroxisome proliferator-activated receptor gamma (PPARγ) and enzymes that drive adipocyte differentiation by activating lipogenesis [164,165]. Concomitantly, E7-expressing preadipocytes accumulate enlarged lipid droplets. This phenotype is exacerbated by oleic acid treatment, which induces TAG synthesis and further increases the number of enlarged lipid droplets, suggesting E7-induced lipogenesis drives lipid droplet accumulation. These findings align with studies showing E7 expression induces AKT signaling and E2F1 expression, which bothincrease lipogenesis [166,167,168].

### 2.4. Latency-Driven Organelle Remodeling and Viral Oncogenesis

The studies reviewed above collectively illustrate the complex relationship between oncovirus-driven organelle remodeling and viral oncogenesis. Gammaherpesviruses directly or indirectly modify mitochondria to reinforce latent cell survival, while HTLV-1 and HPV employ nuanced strategies that can impede latent cell survival. It is important to note that, in the case of HTLV-1 and HPV, the viral proteins p13 and E2, respectively, are known to restrict viral gene expression to maintain viral latency [35,114]. This role in latency maintenance appears to contrast the capacity of both p13 and E2 to promote latent cell sensitivity to apoptosis through mitochondrial fragmentation. However, this approach may be a dedicated immune evasion strategy impairing mitochondrial immune signaling via the MAVS/RIG-I axis during latency [169]. Therefore, reduced latent cell proliferation rates among HTLV-1 and HPV-infected cells may be traded for enhanced immune evasion. For HTLV-1, whether the loss of p13 function in vivo—unleashing HTLV-1 viral gene expression and Tax-mediated oncogenesis—is a critical determinant of poor prognosis for HTLV-1-driven lymphomas remains unknown. For HPV, while E2 promotes apoptosis sensitivity in infected cells, other oncoproteins like E6 and E7 promote pro-survival mitochondrial remodeling [105,119]. This may reflect the temporality of the HPV replication through different tissues, where E2 expression in HPV-latent basal cells sensitizes them to apoptosis, while increased E6 and E7 expression protects cells during keratinocyte differentiation through upper epithelial layers. Thus, there appears to be strong evidence that each oncovirus has acquired strategies to both promote and suppress latency establishment under select environmental conditions, in part through modulating organelle biology.

A clear dichotomy emerges in mitochondrial remodeling between the two human gammaherpesviruses: KSHV latency reduces mitochondrial numbers and promotes fusion in endothelial cells [86,88], whereas EBV latency induces mitochondrial fragmentation across epithelial and B-cell models [34,93,99]. Several factors may explain these contrasting phenotypes. Firstly, cell-type-specific differences may contribute to distinct metabolic requirements for latent cell survival and proliferation or differential activation of MAVS-mediated immune surveillance [169]. Secondly, KSHV and EBV latency proteins appear to interface with mitochondria through different mechanisms: Yogev and colleagues [86] found that several KSHV latency proteins localize directly to mitochondria, whereas EBV studies pointed to pathways that signal indirectly from the plasma membrane. This suggests divergent evolutionary strategies—KSHV latency may have evolved to directly alter mitochondrial membrane composition and respiratory complex function, while EBV leverages plasma membrane-initiated signaling cascades to indirectly tune mitochondrial dynamics. Notably, despite these opposing mitochondrial phenotypes, the downstream metabolic outcomes—including alterations in aerobic glycolysis, fatty acid synthesis, and OXPHOS—often converge between KSHV and EBV infections [170,171]. These observations suggest that distinct mitochondrial remodeling strategies may ultimately serve shared proliferative programs. Resolving these possibilities will require systematic mapping of mitochondrial remodeling across diverse cell types and infection states for both viruses. Also valuable would be the further characterization of how KSHV and EBV latency protein homologs localize to and interface with mitochondrial membranes, and how they influence the mitochondrial proteome and lipidome.

Alongside mitochondria remodeling, several reports of peroxisome and lipid droplet remodeling during oncovirus infection also reveal distinct vulnerabilities in latent cell viability as linked to lipid flux. For KSHV, EBV, and HPV, perturbation of lipid synthesis either directly by targeting FASN or indirectly by targeting PPARγ, a transcriptional regulator of lipid flux, leads to a distinct loss of latent cell viability. This loss in viability can be alleviated via supplementation of downstream fatty acids such as palmitic acid, emphasizing a key reliance on this pathway for cell survival. These findings lead to a critical question: what is the role of fatty acids in maintaining viral latency and/or latent cell proliferation? One clear contribution is the incorporation of fatty acids into membranes throughout the cellular interior and the plasma membrane. Their incorporation directly influences overall membrane composition, membrane fluidity, and signal transduction [172]. Reinforcing the importance of these membrane alterations, remodeling of peroxisomes and lipid droplets has also been tied to increased cell invasiveness [98] and angiogenesis [156], pointing to a link between lipid flux and cancer cell motility. Lastly, alterations in lipid species accumulation during viral latency would also impact the formation and release of extracellular signaling bodies, including exosomes, which have well-characterized roles in viral oncogenesis [173,174]. Furthermore, recent studies have shown that fatty-acid-enriched exosomes can also influence metabolic homeostasis [175] and suppress immune responses involved in anti-tumor immunity [176]. Therefore, modifications in lipid flux as regulated by peroxisomes and lipid droplets may be a strategy by which oncoviruses tune the rate of release of extracellular signals across the virus tumor microenvironment. Collectively, these findings emphasize how changes in lipid metabolism can support nearly every facet of viral oncogenesis and present a unique feature of infection that can be exploited for therapeutic applications. Thus, it is critical that we continue to explore how each oncovirus has evolved to modify metabolic subcellular compartments and the consequences of these remodeling events on oncovirus persistence through latency.

**Table 1:** Reports on organelle remodeling during latent oncovirus infection and its connections to viral oncogenesis.

Virus	Organelle	Cell Type	Remodeling Event	Oncogenic Phenotype	Viral Effectors	Host Targets	Ref
KSHV	Mitochondria	Endothelial (LEC)	Reduced numbers	Increased glycolysis, decreased OXPHOS ^1^	KSHV miRNAs	HIF1α, COX-IV, TFAM, HSP9A, EGLN2	[86]
		Endothelial (TIME), Epithelial (HEK293)	Reduced numbers, increased length, increased transcript levels, increased genome copies	Increased cell survival and proliferation	vFLIP, vCyclin, KapB	N/A	[88]
	Peroxisomes	Endothelial (TIME)	Increased numbers	Increased cell survival and proliferation	Latency locus	ABCD3, ACOX1	[139]
		Epithelial (HEK293), B-lymphocyte (BCBL-1)	Altered membrane composition	Increased cell survival and proliferation	vFLIP	MAVS	[143]
	Lipid Droplets	Endothelial (TIME)	Increased numbers	Increased cell survival and proliferation	N/A	FASN, Palmitic acid	[155]
		Endothelial (HUVEC)	Increased numbers	Increased angiogenesis	N/A	N/A	[156]
EBV	Mitochondria	Epithelial (AGS)	Fragmentation	Increased cell migration, EMT ^2^ induction, chemoresistance	LMP2A	Drp1, Mfn1, Notch, PI3K, AKT	[93]
		Epithelial (CNE1, HONE1)	Fragmentation	Increased cell survival and proliferation, chemoresistance	LMP1	Drp1, AMPK, cyclinB1/CDK1	[99]
		B-lymphocyte (LCLs)	Altered cristae	Increased cell survival and proliferation, decreased OXPHOS ^1^, altered metabolism (1-carbon, aspartate)	EBNA2	MYC, Cardiolipin	[34]
	Peroxisome	B-lymphocyte (LCLs)	Altered biogenesis and β-oxidation	Altered lipid metabolism	N/A	Peroxisome biogenesis factors, lipid importers	[144]
		PMBC, B-lymphocytes (EB3, Daudi)	Altered biogenesis and β-oxidation	Altered lipid metabolism	N/A	Peroxisome biogenesis factors, lipid importers	[146]
	Lipid Droplet	Epithelial (CNE1 and TW03)	Increased numbers	Enhanced cell migration	LMP2A	ATGL	[98]
HPV	Mitochondria	Keratinocytes (C33-A, Saos-2, HaCaT)	Reduced cristae	Increased oxidative stress, increased glycolysis, increased lactate secretion	E2	HIF1α	[112]
		Keratinocytes (UM-SCC-22A, UM-SCC-1A, UM-SCC-47, UPI-SCC-90)	Clustering and fragmentation	Increased cell survival and proliferation	E7	Rb, E2F5, Drp1, Ceramide	[105]
	Lipid Droplet	preadipocytes	Increased numbers, increased volume	Increased cell survival and proliferation	E7	PPARγ	[163]
HTLV-1	Mitochondria	Epithelial cells (HeLa), T-lymphocytes (Jurkat)	Fragmentation, decreased membrane potential, increased membrane permeability, increased mitochondrial K^+^ import	Increased oxidative stress, decreased cell survival and proliferation	p13	N/A	[125]

^1^ OXPHOS: oxidative phosphorylation. ^2^ EMT: endothelial-to-mesenchymal transition.

## 3. Organelle Remodeling During Active Oncovirus Replication

**Figure 2:**
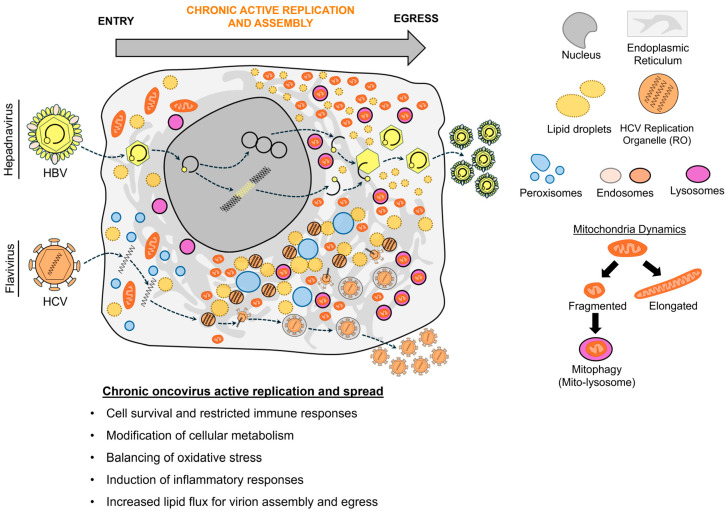
Chronic active oncovirus replication induces organelle remodeling with links to viral oncogenesis: Chronic active replication of hepatitis B virus (HBV) (**top**) and hepatitis C virus (HCV) (**bottom**) temporally regulates organelle dynamics. This schematic shows alterations in organelle structure that support virus replication and spread. Virus replication proceeds from left to right, showing viral entry, replication, assembly, and egress. The legend on the right identifies each organelle and mitochondria remodeling events throughout HBV and HCV infections. Listed are outcomes of viral oncogenesis linked to organelle remodeling events driven by persistent HBV and HCV infections.

While several oncoviruses employ viral latency for persistence, all oncoviruses must undergo active replication to spread. During this phase, oncoviruses systematically remodel subcellular compartments to meet metabolic and signaling demands, while evading, restricting, or co-opting intrinsic immune responses [176]. Latency-establishing oncoviruses (KSHV, EBV, HPV, HTLV-1) undergo an initial round of active replication before transitioning to latency, followed by either reactivation (KSHV, EBV, HTLV-1) or gradual transition back to active replication (HPV). Other oncoviruses, including HBV and HCV, establish persistent infections characterized by chronic low-level active replication, with a strategy of constant non-cytocidal replication (HBV) or rapid spread and immune evasion (HCV). This section highlights how active oncovirus infection remodels organelles, focusing on metabolic remodeling (mitochondria, peroxisomes, lipid droplets) and reshaping secretory compartments for virion assembly and egress (ER, Golgi, endosomes) (Figure 2, Table 2).

### 3.1. Mitochondria Remodeling During Active Oncovirus Infection

While mitochondria support cell survival and proliferation during viral latency, they also play additional roles during active viral replication: activating intrinsic immune responses [177] and inducing cell death to prevent viral spread [178,179]. Released mitochondrial content (mtDNA, mtROS) further potentiates these antiviral processes in infected and neighboring cells [180,181,182,183]. However, alongside these antiviral roles, mitochondria are also modified during active viral replication to meet the metabolic requirements of viral gene expression and virion production [49,53,184]. Consequently, oncoviruses have evolved to target mitochondria both to rewire metabolism for replication and to suppress immune signaling. This section examines how oncoviruses balance mitochondrial respiration and immune responses during active replication.

#### 3.1.1. Mitochondria Remodeling During HBV Infection

Hepatitis B virus (HBV), an enveloped dsDNA hepadnavirus, causes chronic liver disease, liver failure, and hepatocellular carcinoma (HCC) [17]. HBV-driven HCC exhibits metabolic dysregulation, including the above-mentioned Warburg metabolism, oxidative stress, and inflammation-induced immune exhaustion [185,186,187]. The HBV oncoprotein HBx drives HCC progression through multiple pathways including: cell growth, lipogenesis, inflammation, immune evasion, altered bioenergetics, angiogenesis, and apoptosis impairment [188]. A key oncogenic function of HBx is targeting mitochondrial dynamics [32,189,190,191,192,193,194]. HBx consistently induces mitochondrial fragmentation following HBV infection (HepG2.2.15, HepAD38) [32,194] or HBx expression alone (HuH7, HepG2) [32,191,194]. Kim and colleagues first showed that HBx causes perinuclear mitochondrial clustering and translocation of active Drp1 (i.e., phosphorylated at Ser616) via cyclin B/CDK1 phosphorylation [32], which is the same pathway co-opted by EBV LMP1 in latent B cells [99]. HBx also upregulates mitophagy factors (Parkin, PINK1, LC3B) that deliver fragmented mitochondria to lysosomes for degradation. Disrupting Parkin-mediated mitophagy induces apoptosis in HBV-infected and HBx-expressing hepatocytes. These findings demonstrate that HBx-induced mitochondrial fission and turnover promote infected cell survival. Interestingly, the severity of HBx-induced mitochondrial fission depends on genotype-level variations in HBx [191], linking HBx-mediated mitochondrial remodeling to virus genotype-specific pathogenesis outcomes [195]. This extends not only to physical remodeling of mitochondria but also to the differential impact of HBx variants on mitochondrial functions, such as cytochrome c oxidase activity and membrane potential. However, despite these variations in the extent of remodeling, all HBx variants cause mitochondrial structural changes that increase oxidative stress and pro-inflammatory responses in a genotype-dependent manner. Collectively, these studies demonstrate that HBV-encoded HBx targets multiple dimensions of mitochondria biology, with connections between genetic variation among HBV strains, the degree of mitochondria remodeling, and potentially HBV-driven HCC outcomes.

#### 3.1.2. Mitochondria Remodeling During HCV Infection

Like HBV, HCV establishes persistent active replication in the liver, causing chronic hepatitis, cirrhosis, and hepatocellular carcinoma [196]. However, unlike HBV infection, HCV infection itself does not directly transform the host cell. Instead, active HCV replication indirectly contributes to the oncogenic tumor microenvironment by perpetuating oxidative stress. This excessive oxidative stress in turn triggers chronic inflammation, dysregulation of intra- and intercellular signaling pathways (i.e., Wnt/β-catenin, PI3K/Akt), and promotes EMT-driven metastasis [197,198,199,200,201]. One major contribution to these phenotypes is HCV-driven mitochondrial remodeling, which promotes changes in cellular metabolism, oxidative stress, chemoresistance, and immune evasion [202,203,204,205,206,207,208]. An early report by Deng and colleagues first showed that HCV infection induces mitochondrial swelling and clustering distinct from the flavivirus “membranous web”, causing reduced membrane potential and oxidative stress from mitochondrial superoxide release [202]. This impaired mitochondrial function was later linked to impaired OXPHOS and a glycolytic shift via HIF-1α stabilization, characteristic of Warburg metabolism [203]. HCV drives mitochondrial fragmentation through increased Drp1 (Ser616 phosphorylation) and MFF levels, with translocation of fragmented mitochondria into perinuclear clusters [204,205]. At this site, fragmented mitochondria undergo enhanced Parkin-dependent mitophagy. Inhibiting Parkin or impairing the expression of fission factors restricts HCV replication, decreasing virion release, ATP generation, and lactate secretion. This reversal of fragmentation/mitophagy also activates interferon-sensitive response element (ISRE)-mediated immune responses, cytochrome c release, and caspase-mediated apoptosis. Collectively, these studies emphasize how HCV-induced mitochondrial fragmentation and turnover impede immune responses and oxidative stress-induced apoptosis. Collectively, this mitochondrial remodeling therein promotes viral replication and persistence in the liver tumor microenvironment.

#### 3.1.3. Mitochondria Remodeling During EBV Lytic Replication

While KSHV and EBV modify host metabolism and immunity during latency, they face many of the same metabolic and immune evasion demands during active lytic replication as HBV and HCV. As such, both gammaherpesviruses have evolved strategies to remodel mitochondria during active lytic replication. Early studies of EBV reactivation from latency in epithelial and B cells showed mitochondrial reorganization into tightly bundled clusters resembling fusion events, intensifying from 24 to 48 h post-reactivation [209]. Mitochondrial bundling correlated with increased membrane potential at 24 h post-reactivation, followed by a decline at 48 h post-reactivation, suggesting an initial respiratory burst followed by a progressive downregulation. These changes coincided with microtubule but not actin reorganization, perhaps indicating a coordination with the formation of the EBV cytoplasmic assembly complex (AC) [210]. Expression of immediate-early lytic proteins BZLF1 and BRLF1 alone partially recapitulated mitochondrial bundling and perinuclear translocation. A later study by Vilmen and colleagues confirmed EBV reactivation-induced mitochondrial clustering, showing that EBV lytic reactivation in epithelial and B cells, or expression of viral Bcl-2 homolog BHRF1 alone, drives mitochondrial fragmentation and aggregation into “mito-aggresomes” [211]. BHRF1 localizes to and promotes mito-aggresome formation by inducing Drp1 expression, while restricting the inhibitory Drp1 (Ser637) phosphorylation. BHRF1-induced fission increases Parkin-dependent mitophagy. As seen with HBV and HCV, inhibiting Drp1 expression impaired BHRF1’s restriction of type I IFN responses via MAVS/STING [211]. Remarkably, fragmentation preserved membrane potential, allowing for sustained OXPHOS despite enhanced fragmentation and mitophagy turnover. These findings demonstrate how EBV lytic replication induces the fragmentation and degradation of mitochondria (mitophagy) to restrict MAVS/STING immune signaling, while paradoxically maintaining mitochondrial OXPHOS.

#### 3.1.4. Mitochondria Remodeling During KSHV Lytic Replication

KSHV lytic replication also induces the remodeling of the mitochondria compartment to support the outcomes of active lytic replication [212,213,214]. Vo and colleagues found that mitochondrial content decreased upon KSHV lytic reactivation in epithelial and B-cell models of reactivation [212]. This effect was linked to the expression of viral interferon regulatory factor 1 (vIRF1), a lytic oncoprotein that drives cellular transformation [215,216,217]. vIRF1 clears mitochondria through direct interaction with the mitophagy receptor NIX via its PD domain, activating NIX-mediated mitophagy. This enhanced mitophagy correlated with mitochondrial–lysosomal clustering termed “mito-lysosomes”, resembling the mito-aggresomes described during EBV lytic replication [211]. Loss of the vIRF1 PD domain increased mitochondrial content but severely reduced lytic cell viability, indicating vIRF1-induced mitochondrial alterations protect cells from mitochondria-mediated cell death. Defective or absent vIRF1 also decreased virion production post-reactivation. Emphasizing its promise as a potential therapeutic target, a small peptide targeting vIRF1’s PD domain impaired vIRF1-induced mitochondrial remodeling and mitophagy. vIRF1 overexpression also drove Drp1-dependent mitochondrial fragmentation, as Drp1 dominant-negative mutation (K38A) reversed vIRF1 fragmentation in epithelial cells. However, the mechanism governing Drp1-mediated fragmentationduring KSHV lytic replication remains unclear. This interface between vIRF1 and mitophagy was further elucidated in a follow-up study showing that vIRF1 directly binds to GABARAPL1 in an LC3-dependent manner via NIX. This interaction activates NIX-mediated mitophagy and promotes productive KSHV lytic replication [213]. These studies demonstrate how enhanced turnover of mitochondria during active KSHV lytic replication is essential for lytic cell survival.

While previous studies showed how KSHV induces mitochondrial turnover during lytic replication, a recent study by Zhu and colleagues revealed that KSHV viral Beclin-2 (vBcl-2, ORF16), a mitochondria-localized transmembrane protein, can also drive changes in mitochondrial architecture to support lytic reactivation [214,218,219,220,221]. The authors reported that vBcl-2 interacts with NDP kinase NM23-H2 to induce severe mitochondrial fission and promote lytic replication [214]. Interestingly, KSHV lytic replication did not affect fusion (Mfn1/Mfn2, Opa1) or fission (Drp1, Mff) factor expression, and Mfn2 overexpression could not reverse vBcl-2-induced fragmentation in iSLK cells. However, depleting Drp1 or MFF reversed fragmentation, while significantly reducing virion production, a phenotype recapitulated by Drp1 inhibitor Mdivi-1 or dominant-negative mutant K38A. This reinforces the importance of post-translational modifications on either Drp1 or MFF that are likely driving this exacerbated fission [99,222]. Unlike HBV or HCV, KSHV-induced fragmentation did not alter membrane potential, mtROS, or mtDNA release. This suggests that these KSHV-modified mitochondria are fully functional despite exacerbated fragmentation and cristae reduction. Furthermore, vBcl-2 expression does not alter PINK1 colocalization with fragmented mitochondria, indicating vBcl-2-induced mitochondria fragmentation is not linked to mitophagy. Therefore, KSHV regulation of mitophagy may be a function dependent on vIRF1 expression [212,213]. To drive this increased fission, vBcl-2 relocalizes NM23-H2 to mitochondria, forming an NM23-H2-Drp1 complex that provides local GTP for Drp1-mediated fission. This interaction significantly restricts IFN-β production in reactivated epithelial and B cells by impeding MAVS cluster aggregation at mitochondrial surfaces., This interaction also blocks MAVS-mediated TBK1 activation and IRF3 dimerization. While not affecting viral gene expression or genome amplification, vBcl-2 function supports late-stage virion assembly and egress by promoting capsid trafficking from the nucleus to the cytoplasm by suppressing interferon-stimulated genes TRIM22 and MxB [214]. A small-molecule inhibitor targeting vBcl-2-NM23-H2 interaction impaired virion production in epithelial and B cells in a dose-dependent manner, without affecting viability, DNA replication, transcription, or protein expression. This inhibitor also prevents vBcl-2-induced mitochondrial fragmentation and restores MAVS-mediated IFN and ISG production to restrict KSHV assembly [214]. In addition to highlighting the effect of KSHV lytic replication on mitochondrial remodeling, this study also points to connections between the different organelle remodeling strategies observed across infection time. Interestingly, the mitochondrial fragmentation and turnover observed during active replication contrast with the mitochondrial elongation seen during viral latency. It is, however, important to note that elongation has only thus far been reported in KSHV-infected endothelial cells, while fragmentation has been shown in lytic epithelial cells.

While mitochondrial remodeling promotes KSHV and EBV lytic replication, Zhang and colleagues recently uncovered a role for mitochondrial dynamics in maintaining the latent-to-lytic switch [223]. Maintenance of the latent-to-lytic switch critically determines viral oncogenesis, as excessive lytic replication promotes immune recognition and clearance of both KSHV and EBV [10,14,,224]. One of the critical triggers for the KSHV and EBV latent-to-lytic switch is excessive oxidative stress, which is in large part regulated by mitochondria dynamics [225,226,227,228,229,230,231]. A recent study by Zhang and colleagues further emphasizes this connection between excessive ROS and latency maintenance. The authors found that loss of mitochondria-localized transforming growth factor-beta regulator 4 (TBRG4) protein in KSHV-infected epithelial, endothelial, and B cells, as well as EBV-infected epithelial GC cells, induced KSHV/EBV lytic replication [223]. The loss of TBRG4 decreases mitochondrial OXPHOS and increases hydrogen peroxide levels, reflecting an induction of mtROS release known to trigger the transactivation of lytic gene promoters [225,232]. Treatment of cells depleted of TBRG4 with ROS scavengers mitigates the increase in mtROS and restricts KSHV and EBV lytic reactivation from latency, suggesting that TBRG4 restricts ROS production at mitochondria to maintain viral latency. This study highlights how mitochondrial function is not only vital to the outcomes of viral latency and lytic replication but also can contribute to the maintenance of the pathways that regulate the switch between the two states of oncovirus infection.

### 3.2. Peroxisome Remodeling During Active Oncovirus Infection

**Figure 3:**
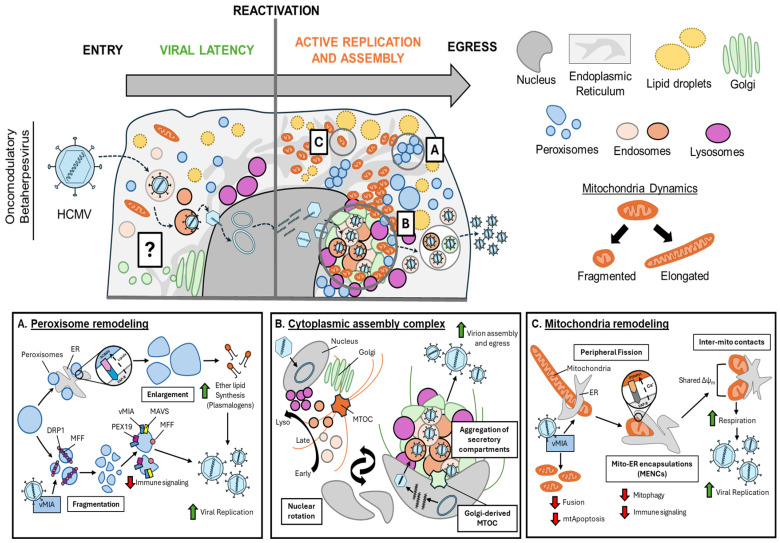
Infection with the oncomodulatory virus HCMV alters organelle structure–function relationships. The oncomodulatory betaherpesvirus human cytomegalovirus (HCMV) induces temporally tuned alterations in organelle structure and organization during its lytic replication cycle, thereby dysregulating cellular respiration, lipid metabolism, and immune signaling, and promoting virus replication and spread. (**A**) HCMV-encoded pUL37x1 promotes peroxisome fragmentation through the functions of fission factors Drp1 and MFF. During active replication, peroxisome numbers increase, and altered peroxisome morphology, resembling enlargement, is observed late in infection. These remodeling events have been linked to the inhibition of immune signaling early in infection and the elevation of ether lipid synthesis (plasmalogens) needed for HCMV secondary envelopment late in infection. (**B**) During late stages of HCMV replication, membranes derived from multiple secretory organelles (ER, Golgi network, and endolysosomes) are aggregated to the perinuclear space to form the cytoplasmic virion assembly complex. The herpesviral assembly complex rests upon a concentrated microtubule organization center (MTOC) that coordinates membrane organization, virion maturation, and virion egress. The formation of the HCMV assembly complex relies on rotation and remodeling of the nucleus into a “kidney-bean” shape. (**C**) HCMV-encoded pUL37x1 (viral MIA (vMIA)) promotes mitochondrial fission and inhibits mitochondrial apoptosis (mtApoptosis). Following peripheral fission, the fragmented mitochondria display altered contacts with the ER (endoplasmic reticulum), forming mitochondria–ER encapsulations (MENCs). MENC structures inhibit mitophagy and retain the bioenergetic activity of mitochondria. MENCs also promote inter-mitochondrial contacts that allow for the sharing of membrane potential (∆ψ_m_). (?): Currently, there are no studies documenting organelle remodeling during HCMV latency.

During active viral replication, oncoviruses induce alterations in cellular respiration, lipid metabolism, ER stress, and mitochondrial dysfunction that increase ROS accumulation and oxidative stress [27,30,132]. This excessive oxidative stress not only damages the host cell, but can also promote cellular transformation across the tumor microenvironment [27,29,185]. To balance this stress, the virus and host rely in part on peroxisomal antioxidant functions through the activity of peroxisomal catalase and peroxiredoxins [233,234] (Figure 2). Peroxisomes are also well known for their roles in immune signaling through coordination of MAVS oligomerization at peroxisome membranes, a process restricted by HCMV and HSV-1 [235,236,237]. However, peroxisomes were also shown to play pro-viral roles through their coordination of plasmalogen synthesis (i.e., ether lipids), which was shown to support virion assembly for enveloped viruses like HCMV (Figure 3A), influenza, and West Nile virus (WNV) [47,48,234,238,239]. This lipid synthesis function is also thought to contribute to oncovirus replication through roles in membrane integrity, fluidity, and fission–fusion events [240]. Given these diverse functions, the contributions of peroxisomes during active oncovirus replication or to the outcomes of viral oncogenesis are currently under active investigation.

Supporting that peroxisomes are remodeled during active oncovirus replication, a study by Fourchambault and colleagues found that HCV infection of hepatocytes triggers peroxisome redistribution to virus replication complexes woven with ER membranes [31,241]. This recruitment coincides with the redistribution of peroxisomal VLCFA importer ACBD5 to HCV replication organelles. However, ACBD5 overexpression did not induce peroxisome localization near replication organelles, and ACBD5 knockout (i.e., losing ER–peroxisome tethering with VAP-B) [242,243] did not affect HCV replication or peroxisome redistribution. Hence, the altered ACBD5 localization does not contribute to the peroxisome association with virus replication compartments. HCV infection also impacts peroxisome morphology and numbers, increasing volume while decreasing numbers, resulting in fewer but enlarged peroxisomes near replication sites. Despite these remodeling events, functional peroxisome loss (via PEX5 knockout) or complete peroxisome loss (via PEX3 knockout) did not impact HCV replication, and VLCFA beta-oxidation remained unchanged, indicating that peroxisomal lipid metabolism does not significantly contribute to HCV replication. However, HCV replication does increase ROS accumulation specifically in peroxisomes. This ispossibly due to a defect in catalase function, as its expression remains unchanged during HCV replication. These results suggest that HCV-induced peroxisomal remodeling causes ROS accumulation in enlarged peroxisomes, which does not seem to directly impact the outcomes of HCV replication. However, given peroxisomal ROS accumulation, investigating its contribution to oxidative stress across the HCV-induced tumor microenvironment is warranted, as peroxisomal changes (number, composition) have been linked to hepatocellular carcinoma [135].

### 3.3. Lipid Droplet Remodeling During Active Oncovirus Infection

While lipid droplets primarily store and metabolize lipids to support cell survival and proliferation during viral latency, they are also co-opted during active replication to support virion maturation of enveloped oncoviruses. These alterations in lipid synthesis and flux are especially important for HBV and HCV, as lipid flux in hepatocytes critically affects both viral replication and hepatocellular carcinoma development [166,244,245].

#### 3.3.1. Lipid Droplet Remodeling During HBV Infection

HBV infection was shown to alter lipid metabolism and lipid droplet dynamics in support of viral replication [246,247,248,249,250,251,252,253]. The HBV HBx protein is a primary driver of lipid droplet remodeling through multiple mechanisms. HBV infection and HBx overexpression in hepatocytes upregulate fatty acid binding protein 1 (FABP1), increasing intracellular fatty acid uptake and lipid droplet accumulation in hepatocytes [247]. HBx also induces lipogenic factors (LXR, SREBP1, PPARγ) that enhance lipogenic enzyme expression and lipid droplet formation [254,255,256,257]. However, while HBV infection increased lipid droplet numbers, Yasumoto and colleagues found that infected cells tend to have smaller lipid droplets due to the downregulation of CIDEB and CIDEC, which are critical for HBV replication [248]. In parallel to changes in lipid droplet number and size, HBV infection restricts autophagy-mediated lipid droplet turnover via PI3K/AKT and mTOR signaling [250]. Excitingly, recent work by Chowdhari and colleagues has demonstrated that an HBV-encoded viral miRNA, HBV-miR-3, highly expressed in chronic HBV patients, downregulates cholesterol efflux regulator ABCA1, increasing cholesterol and lipid droplet accumulation [253]. Expression of HBV-miR-3 also promotes several downstream oncogenic phenotypes, such as cellular proliferation and colony formation (reversible with cholesterol-lowering drugs) [253]. Collectively, these studies highlight how HBV infection drives alterations in lipid metabolism and lipid droplet remodeling through direct (HBx, HBV-miR-3) and indirect (PI3K/Akt/mTOR) mechanisms to support virion maturation and infectivity [252].

#### 3.3.2. Lipid Droplet Remodeling During HCV Infection

Following primary infection, HCV viral proteins embed in the ER lumen, which is then remodeled to form replication organelles (ROs) as double-membrane vesicles that shield viral replication from immune surveillance [258,259,260,261]. Double-membrane vesicles form through two pathways: vesicles bud and wrap back on themselves, or membrane protrusions curl back while connected to the parent membrane [262]. These processes iteratively create a flavivirus membranous web. Lipid droplet accumulation at ROs, triggered by viral genome recognition by DDX3X and lipid droplet stabilization by the HCV core protein [263,264,265,266,267], is essential for membranous web formation and virion maturation. Trafficking of HCV proteins to ROs and connecting them to lipid droplet membranes requires host factors DGAT1 and RAB18, which bridge lipid droplet and replication organelle membranes [268,269]. A synchronous interaction between NS5A, RAB18, and the lipid droplet–membrane perilipin PLIN3 forms RO–lipid droplet contact sites critical for genome amplification and virion maturation [270,271,272]. These studies highlight lipid droplets’ critical role throughout HCV replication, from RO–lipid droplet contact formation to virion production. This lipid droplet accumulation may contribute to HCV-induced steatosis, which promotes excessive inflammation across hepatocellular carcinoma tumors [245,267,273].

### 3.4. Remodeling of Secretory Compartments During Active Oncovirus Infection

Virion assembly and egress are critical for the spread of an infection. For enveloped oncoviruses (HBV, HCV, KSHV, EBV, HTLV-1), while capsid assembly occurs in the nucleus or cytoplasm depending on the virus, final virion maturation requires the acquisition of host membranes from secretory organelles (PM, ER, Golgi, endolysosomes). This process can involve the repositioning of non-secretory organelles (lipid droplets, peroxisomes, mitochondria) near virion assembly sites to support the final steps of the replication cycles, as seen with lipid droplets and HCV ROs. Therefore, oncoviruses must interface with and alter the organization and/or lipid composition of cellular membranes to facilitate assembly and egress. This manifests as systemic reorganization of secretory organelles to form cytoplasmic replication organelles (ROs), as with HCV, or viral assembly complexes (ACs), as with KSHV and EBV. Having discussed HCV ROs previously, this section focuses on how KSHV and EBV form cytoplasmic assembly sites and how these structures contribute to viral oncogenesis.

The cytoplasmic assembly complex, once considered exclusive to the betaherpesvirus HCMV, comprises remodeled ER, Golgi, and endolysosomal membranes that support secondary envelopment, tegumentation, and virion egress (Figure 3B) [274,275,276,277,278,279,280,281,282,283,284]. For HCMV, assembly complex formation is an intricate process, involving the recruitment of viral proteins, remodeling of the nucleus into a “kidney-bean” shape, and nuclear rotation [285]. Like HCV, HCMV-induced aggregation of host membranes and proteins to the assembly complex supports viral replication and activates oncogenic signaling pathways. These include HCMV-induced alterations in mTOR distribution that promote metabolic rewiring [286,287,288,289,290]. However, emerging studies support that assembly complex formation is shared between the betaherpesvirus HCMV and gammaherpesviruses KSHV and EBV across multiple cell types [210,291,292,293]. A study by Dai and colleagues demonstrated that EBV lytic reactivation remodels nuclear and secretory organelles to form a cytoplasmic assembly complex in both epithelial and B-cell models [210]. Early during lytic replication, the viral kinase BGLF4 and late viral proteins (BBLF1, gp350, gp110) condense at a perinuclear concave space, coupled with nuclear remodeling into the characteristic “kidney-bean” shape. These viral proteins cluster with host membranes, including the Golgi, and a restructured microtubule organization network (MTOC), reminiscent of the HCMV assembly complex. This coincides with the redistribution of cytoskeleton protein IQGAP1 to the EBV assembly complex. Notably, while ER membranes are absent, early endosomes and lysosomes accumulate at the assembly complex center. BGLF4 kinase alone drives MTOC alterations and organelle reorganization, though not to the extent seen during infection, suggesting that additional viral proteins are required. IQGAP1 aggregates at the EBV assembly complex during mid-stage replication but relocates to the plasma membrane during late-stage replication. While IQGAP1 knockdown does not prevent assembly complex formation, it significantly impedes virion egress. This study demonstrates the critical role of the viral kinase BGLF4 in EBV assembly complex formation.

Just as with EBV, KSHV-encoded viral proteins have been shown to coordinate the formation of the KSHV cytoplasmic assembly complex. Early studies of the tegument protein KSHV ORF45 have shown it to be a critical piece of productive KSHV lytic replication [294]. This includes ORF45-mediated immune evasion, through inhibition of type I interferon responses [295], and essential roles in promoting viral late gene expression, virion production, and virion infectivity [295,296]. These studies link ORF45 to virion maturation and egress. In line with this, following capsid packaging and tegumentation, ORF45 was shown to mediate the interaction between newly made capsids and the KSHV tegument, forming a complex that is then trafficked by the cargo-binding protein KIF3A [297]. ORF45 localization to lipid rafts, requiring mono-ubiquitination, is essential for viral maturation and release [292]. Studies also show that ORF45 drives the reorganization of Golgi, lysosomes, and lipid rafts to facilitate virion maturation and egress. However, whether KSHV forms a fully fledged cytoplasmic assembly complex like HCMV and EBV remains unclear. Recent work by Zhou and colleagues provides strong evidence for KSHV assembly complex formation, showing that ORF52, a DNA-binding tegument protein that restricts cGAS activity [298], can also induce assembly complex formation through liquid–liquid phase separation properties [291], though this was shown outside the context of KSHV lytic replication.

These studies demonstrate that KSHV, EBV, and HCV remodel cytoplasmic compartments to support virion assembly, maturation, and egress. The formation of these expansive structures imposes significant physical and resource stress on cells. While factors coordinating membrane and host protein selection to ROs or ACs remain unclear, the altered flow of metabolites (lipids) and proteins (IQGAP1) has clear consequences. Temporal transitions of host and viral factors localizing to and leaving ACs alter their interactomes and functions, supporting viral infection, while activating oncogenic pathways. Given the potential contribution of phase separation to KSHV and EBV assembly complex formation, non-membrane-bound compartments like cytoplasmic RNA granules may be similarly impacted. Several studies show that KSHV and EBV early lytic replication alters processing bodies (P-bodies) and stress granules (SGs) through complex interactions between host and viral RNA-binding proteins [299,300,301]. These observations suggest that early disassembly of these complexes may enable the redistribution of factors promoting formation of phase-separated viral compartments during late-stage replication.

### 3.5. Organelle Remodeling During Active Oncovirus Replication and Viral Oncogenesis

The induction of mitochondrial fragmentation is a conserved feature of active oncovirus infections across viral families and cell types. Through this mitochondrial remodeling, oncoviruses alter cellular respiration to support replication, restrict MAVS/STING immune signaling, promote oxidative stress, and prevent cell death. For latent oncoviruses, like KSHV and EBV, altering mitochondrial composition appears essential for restraining the latent-to–lytic switch [223]. However, mitochondrial fission induced by several oncoviruses also enhances mitophagy, rapidly clearing mitochondrial content [32,191,193,195,202,203,204,205,211,212,213,214]. Impeding mitophagy or fission machinery in each of these contexts triggers immediate innate immune signaling and, in some models, apoptosis [32,211,212]. This suggests that oncoviruses must balance mitochondrial elongation/biogenesis to maintain stable populations. Simultaneously, induction of mitophagy clears fragmented mitochondria to avoid excessive cytoplasmic ROS accumulation, cytochrome c release, caspase activation, and mitochondrial calcium store release [302]. These effects collectively are exacerbated when fragmentation leads to mitochondrial membrane depolarization. Remarkably, KSHV and EBV lytic replication do not disrupt mitochondrial membrane potential or respiration, unlike HPV, HBV, and HCV [211,214]. This suggests that gammaherpesviruses sustain fragmented mitochondrial function while enforcing increased fission and mitophagy. Sustained membrane potential allows KSHV and EBV to exploit mitochondrial respiration during replication, while avoiding excessive leakage of apoptotic products and ROS during their relatively long replication cycles. This raises two questions: (1) Have oncogenic herpesviruses evolved distinct mechanisms to sustain mitochondrial integrity despite fission and mitophagy? (2) Do cytotoxic products released from depolarized mitochondria benefit the replication and/or spread of oncoviruses despite the accumulating damage to the host cell?

**Table 2:** Reports on organelle remodeling during active oncovirus infection and its connections to viral oncogenesis.

Virus	Organelle	Cell Type	Remodeling Event	Oncogenic Phenotype	Viral Effectors	Host Targets	Ref
HBV	Mitochondria	Hepatocytes (HepG, HepG2.2.15, HepAD38, HuH7)	Fragmentation, increased mitophagy	Increased cell survival and proliferation	HBx	Drp1, cyclinB1/CDK1, Parkin, PINK1, LC3B	[32,194]
		Hepatocytes (HepG2, HuH7)	Swelling, fragmentation, decreased membrane potential	Increased cell survival and proliferation, increased oxidative stress, increased inflammation	HBx	VDAC3	[191]
	Lipid Droplets	Hepatocytes (HepG2)	Increased numbers, reduced volume	Increased viral replication and spread, induces steatosis	HBx, HBV-miR-3	FABP1, ABCA1, LXR, SREBP1, PPARγ, CIDEB, CIDEC, Pi3K/Akt/mTOR	[247,253]
HCV	Mitochondria	Hepatocytes (HuH7)	Swelling and clustering, decreased membrane potential, cytochrome C release	Increased cell survival and proliferation, increased oxidative stress	N/A	BAX, caspases	[202]
		Hepatocytes (HuH7)	Fragmentation, increased mitophagy	Increased viral replication and spread, decreased OXPHOS ^1^, increased glycolysis, increased lactate secretion	N/A	HIF1α, Drp1, MFF, Parkin, caspases	[203,204,205]
	ER, Peroxisome, Lipid Droplets	Hepatocytes (HuH7)	Replication organelle	Increased viral replication and spread, induces steatosis	Core, NS5A	DGAT1, DDX3X, RAB18, PLN3	[263,264,265,266,267,268,269,270,271,272]
KSHV	Mitochondria	B-lymphocytes (BCBL-1), Epithelial (iSLK)	Clustering and fragmentation, increased mitophagy	Increased cell survival and proliferation	vIRF1	NIX, Drp1	[211]
		B-lymphocytes (BCBL-1), Epithelial (HeLa.Kyoto, iSLK)	Clustering and fragmentation, increased mitophagy	Increased cell survival and proliferation	vIRF1	NIX, ATG8, GABARAPL1, LC3B	[213]
		B-lymphocytes (BCBL-1), Epithelial (iSLK)	Clustering and fragmentation, increased mitophagy, reduced cristae	Increased viral replication and spread, restricted immune response	vBcl-2	NM23-H2, Drp1, MFF, PINK1, MAVS, TRIM22, MxB	[214]
		B-cell (BCBL-1), Epithelial (iSLK), Endothelial (HUVEC)	N/A	Maintenance of latency, restricted oxidative stress, enhanced respiration	N/A	TBRG4	[223]
	ER, Golgi, Endosomes	Epithelial (iSLK, COS7)	Cytoplasmic assembly complex	Increased viral replication and spread	ORF45, ORF52	KIF3A, lipid rafts	[297]
EBV	Mitochondria	Epithelial (D98-HE-R1), B-lymphocytes (D98-HE-R1, Raji)	Clustering and fusion, increased membrane potential	Increased viral replication and spread	BZLF1, BRLF1	N/A	[209]
		Epithelial (HeLa, HEK293), B-lymphocytes (Akata)	Clustering and fragmentation, increased mitophagy	Enhanced cell survival and proliferation, restriction of immune response	BHRF1	Drp1, Parkin	[211]
		Epithelial (AGS)	N/A	Latency maintenance	N/A	TBRG4	[223]
	ER, Golgi, Endosomes	Epithelial (TW-01), B-lymphocytes (Akata)	Cytoplasmic assembly complex	Increased viral replication and spread	BGLF4	IQGAP1, MTOC	[210]

^1^ OXPHOS: Oxidative Phosphorylation.

Addressing the first question, the oncomodulatory herpesvirus HCMV, a distant relative of KSHV and EBV, induces severe mitochondrial fragmentation during lytic replication [76,77,79,81] (Figure 3). However, contrary to the fragmentation–dysfunction paradigm, HCMV-induced fragmentation elevates respiration and suppresses apoptosis. HCMV also induces a Warburg-like metabolism, in which both aerobic glycolysis and increased OXPHOS are observed. While the direct association of fragmented mitochondria with elevated respiration in KSHV and EBV infections remains unclear, partial parallels suggest all three viruses sustain mitochondrial integrity despite fragmentation. For HCMV, the viral protein pUL37x1, also known as viral MIA (vMIA), was shown promote mitochondrial fission [77]. This HCMV-induced mitochondrial fragmentation is alsoinhibitsmitochondrial apoptosis (mtApoptosis) [76]. Our group further showed that HCMV infection induces fragmentation by elevating mitochondrial peripheral fission and suppressing fusion [243]. The resulting fragmented mitochondria progeny are then sequestered in ER pockets, forming mitochondria–ER encapsulation structures (MENCs) (Figure 3C) [33,243]. The formation of MENCs is facilitated by membrane contact sites, contacts formed between membrane-bound proteins that coordinate organelle-organelle communication throughout the cell [303]. During HCMV infection, contacts formed between the mitochondrial PTPIP51 and the ER-resident VAP-B [243] form these encapsulations, protecting mitochondria from mitophagy. The fragmented mitochondria further establish inter-mitochondrial contacts, sustaining elevated respiration rates. Of note, MENC-mediated stabilization of fragmented mitochondria also occurs in metastatic melanoma cells, in the absence of an HCMV infection. It remains to be seen whether a similar mechanism for retaining the bioenergetics of fragmented mitochondria is also at play for the oncoviruses KSHV and EBV. Further work is needed to understand whether organelle–organelle contacts can explain divergent mitochondrial fission consequences across oncovirus families.

Addressing the second question, studies by Kim and colleagues on HCV [204,205], and Chen and colleagues on HBV linking HBx-induced mitophagy to increased glycolysis [304] suggest both viruses benefit from accumulating defective mitochondria. Mitochondrial defects then appear to drive aerobic glycolysis, meeting the energetic demands of replication while enforcing cell survival and proliferation. Increased oxidative stress in liver tissue may also benefit HBV/HCV replication [185,305,306]. Understanding mechanisms by which HBV/HCV induce mitochondrial fragmentation will help determine which downstream consequences benefit or impair replication.

Throughout this section, we have also highlighted how oncoviruses drive metabolic reprogramming, including aerobic glycolysis that results in elevated lactate production and secretion from infected cells. For years, increased lactate production was viewed as a metabolic byproduct with no functional consequence. However, recent reports show that secreted lactate plays critical roles in cancer progression and viral infection. During cancer progression, lactate secretion impacts multiple processes across the tumor microenvironment. These functions include intra- and intercellular signaling between cancer and immune cells, exacerbating inflammatory pathways, increasing oxidative stress, and providing a metabolite for energy production in neighboring cancer cells as a TCA cycle substrate (reviewed recently [65]). Furthermore, Zhang and colleagues demonstrated that lactate can be added to proteins as a post-translational modification (PTM), lysine lactylation [307]. Initially recognized for its contribution to epigenetic histone modifications, it is now understood that lysine lactylation can be found across the human proteome and within multiple subcellular compartments, impacting processes linked to cancer progression including genomic stability, cellular respiration, immune responses, and cell differentiation [66,308,309,310,311,312,313,314,315].

During viral infection, the oncomodulatory viruses HCMV and HSV-1, which also induce a Warburg-like metabolism [288,316,317,318], have been shown to alter the host cell lactylation landscape [66]. Lysine lactylation was found to be enriched in intrinsically disordered regions (IDRs) of viral and host proteins and shown to exhibit both anti- and pro-viral effects. Lactylation of the HCMV pUL112-113, known to be critical for orchestrating HCMV nuclear replication compartment formation [319], inhibited the liquid–liquid phase separation needed for virus replication. However, a common feature of HCMV and HSV-1 infected cells was the lactylation of host immune factors, which involved the alanyl-tRNA synthetase (AARS1) enzyme [66]. The lactylation of the nuclear DNA sensor interferon-γ-inducible protein 16 (IFI16) was shown to inhibit its ability to recruit the DNA damage response kinase DNA-PK to sites of viral genome deposition. The inhibiton of IFI16 recruitment therein suppresses immune signaling in response to both HCMV and HSV-1 infections [66]. Indeed, this restriction of immune signaling extends across a virus microenvironment, as uninfected cells neighboring HCMV-infected cells also exhibit decreased immune signaling and increased susceptibility to viral infection [320]. Excessive lactate secreted from HCMV-infected cells may be one of the contributing factors to these inhibited immune responses. Additionally, the uninfected neighboring cells had markers of genomic instability, suggesting a link to the oncomodulatory properties of HCMV [321]. This critical effect of protein lactylation is supported by Yan and colleagues, who showed that lactylation of N-acetyltransferase 10 (NAT10) at K290 by ATAT1 promotes KSHV lytic replication by enhancing KSHV lytic transcript translation efficiency [322]. Remarkably, NAT10 lactylation by ATAT1 is directly promoted by the viral long-noncoding RNA (lncRNA) KSHV polyadenylated RNA (PAN) [130,131], which mediates tRNA acetylation to promote viral gene translation. These studies of infection-driven lactylation and mitochondrial remodeling during oncomodulatory virus (HCMV) and oncovirus (KSHV) infections provide a strong foundation for investigating oncovirus-driven mitochondrial remodeling, induction of aerobic glycolysis, connections to the outcomes of productive viral replication, and how these phenotypes collectively contribute to viral oncogenesis.

## 4. Concluding Remarks and Future Directions

Throughout this review, we have discussed how human oncoviruses remodel organelles to support infection, cellular transformation, and promote the formation of a viral tumor microenvironment. Remodeling varies from subtle membrane composition shifts to changes in organelle size and distribution, with each event tailored to specific infection state demands. This is evident when comparing viral latency and active replication, which represent vastly different cellular states with distinct infection requirements. During latency, oncoviruses coordinate organelle alterations supporting host cell survival, proliferation, and viral genome maintenance. During active replication, organelle remodeling supports cell survival while also meeting the high metabolic demands of replication, coordinating virion maturation, and suppressing immune responses. However, this paradigm is nuanced. Some organelle changes, as with HTLV-1 and HPV, induce excessive oxidative stress and sensitize cells to apoptosis. HCV peroxisome remodeling during active replication contributes solely to redox homeostasis, without influencing infection progression. Additionally, many studies investigated single viral proteins in the absence of infection or showed variation in remodeling extent depending on the genotype or cell type. Therefore, we must continue integrating data across infection models, cell types, and in vivo systems to understand how organelle dynamics exacerbate viral oncogenesis. Remarkably, organelle remodeling patterns are highly conserved across five distinct viral families, multiple cell tropisms, and oncogenesis routes. From mitochondrial fission induction during latent and lytic states to lipid droplet and peroxisome accumulation, oncovirus-driven remodeling exemplifies synergistic evolution with non-viral cellular transformation patterns. Moving forward, it would be valuable to distinguish remodeling occurring in response to oncovirus persistence (indirect oncogenic stress) from direct viral induction sustaining persistence. This is challenging given the deeply intertwined relationship between cellular metabolism and organelle dynamics, where metabolic changes trigger cascades affecting organelle biology and vice versa. Therefore, we cannot take remodeling events at face value; changes in mitochondrial form and composition do not immediately indicate dysfunction. Multi-disciplinary analyses are needed to understand how oncovirus infection induces organelle remodeling, its consequences, and direct contributions to viral oncogenesis phenotypes. While this review focused on six of the seven classified oncoviruses, oncomodulatory viruses like HCMV and HIV-1, though unable to promote oncogenesis alone, can drive oncogenic processes [321,323]. HIV-1 infection-driven AIDS is a primary risk factor for KSHV and EBV-associated cancers, including Kaposi’s sarcoma and B-cell lymphomas [22]. Like HCMV, HIV-1 induces organelle remodeling, particularly of the plasma membrane, to promote viral replication [324]. As such, investigations of oncomodulatory viruses remain exceptionally informative for understanding mechanisms driving organelle alterations and how they promote oncogenic phenotypes.

Looking ahead, the findings highlighted in this review point to important future questions. A challenging question is which of these organelle remodeling events represent host adaptations rather than virus-driven processes. Host-driven organelle remodeling in response to infection is well-documented, including mitochondrial fission/fusion [325,326], phagocytosis [327], and increased lysosome biogenesis [328]. The organelle morphology and composition observed during viral latency or active oncovirus replication, therefore, likely reflects the combined output of both host- and virus-driven pathways. Parsing these contributions remains difficult, including which of these organelle remodeling events are antiviral responses or cell survival strategies co-opted by oncoviruses to promote persistent infection. A related question is how far these remodeling events extend across the tumor microenvironment, such as within uninfected cells proximal to oncovirus-infected cells. Mitochondrial and lipid droplet remodeling during HCV and HBV infections exemplify membrane alterations that potentiate oxidative stress and inflammatory signaling known to drive hepatocellular carcinoma progression [32,191,202,247,263]. HCV-induced hepatocellular carcinoma is a striking case to this point, as the tumor is predominantly composed of uninfected cells transformed by chronic proximal HCV infection [273]. Yet, whether or how neighboring uninfected cells remodel their organelles in response to virus-induced environmental stressors—and how these changes contribute to tumor progression—remains unclear. Addressing these questions will require multidisciplinary approaches that map the nucleic acid, proteomic, and lipid landscapes of organellar compartments during oncovirus infection. Such efforts can build a more comprehensive understanding of how host cells are rewired to combat or support states of viral infection and, critically, how these pathways can be targeted to impede both infected and uninfected cells within tumors. Encouragingly, studies mentioned above demonstrate that therapeutics targeting host or viral factors involved in virus-induced organelle remodeling can selectively kill transformed cells, combat chemoresistance, and alleviate oxidative stress and inflammation. We hope that this review will inspire future investigations of organelle remodeling at the interface of viral infections and cancer development.

## Data Availability

No new data were created or analyzed in this study.

## Abbreviations

The following abbreviations are used in this manuscript:

EBV	Epstein–Barr virus
EMT	Endothelial-to-mesenchymal transition
HCMV	Human cytomegalovirus
HCV	Hepatitis C virus
HPV	Human papillomavirus
HSV-1	Herpes simplex virus-1
KSHV	Kaposi’s sarcoma-associated herpesvirus
OXPHOS	Oxidative phosphorylation
